# Comparative Sonographic Studies of the Urogenital Tract in Lizards

**DOI:** 10.1111/vru.70075

**Published:** 2025-08-16

**Authors:** Nils B. Klützow, Volker Schmidt

**Affiliations:** ^1^ Clinic for Birds and Reptiles, Faculty of Veterinary Medicine University of Leipzig Leipzig Germany

**Keywords:** ultrasound, reptile, diagnostic imaging, kidney, testis, ovary

## Abstract

The assessment of the urogenital tract is of great importance in the diagnosis of diseases in lizards, and sonographic examination is the most suitable imaging method for this purpose. However, reference data are not available for most of the commonly kept species. The objective of this prospective anatomical analytical study was to sonographically examine the urogenital tract of four of the most commonly kept lizard species and compare their kidneys, testes, and ovaries. A total of 41 lizards, 14 bearded dragons (*Pogona vitticeps*), 15 leopard geckos (*Eublepharis macularius*), seven veiled chameleons (*Chamaeleo calyptratus)*, and five panther chameleons *(Furcifer pardalis*) were included. An 8–18 MHz field hockey stick transducer and a 6–15 MHz linear transducer were used. Sagittal and transverse images of the organs were recorded. The kidneys could be visualized and assessed in full length in all lizards, the testes in 18 of 19, and the ovaries in 13 of 22 lizards. The results of the current study could serve as reference values for future studies on the species mentioned.

## 1 Introduction

Reptiles such as bearded dragons (*Pogona vitticeps*), leopard geckos (*Eublepharis macularius*), veiled chameleons (*Chamaeleo calyptratus*), and panther chameleons (*Furcifer pardalis*) are popular lizards in human care, which are regularly presented in veterinary practice.

Diseases of the urogenital tract are of particular relevance. In chameleons, noninfectious kidney disease is the second most common cause of death, and noninfectious reproductive disease is the third most common cause [1]. Clinical signs can be difficult to recognize, especially in the early stages of the disease [2], and as usually only nonspecific clinical signs are shown [3]. Sonographic examination is a noninvasive, rapid, and effective imaging technique for the diagnosis of renal disease in reptiles, but specialized knowledge and experience are required to adequately assess sonographic images due to the anatomical differences between reptile species [4]. In the examination of the reproductive tract in lizards, sonography is superior to conventional radiography [5] and is of great importance in the assessment of the female genital tract of reptiles, such as the evaluation of ovarian activity and the detection of pregnancy [6, 7]. To date, there are only a few studies dealing with the sonography of the urogenital tract of the mentioned lizard species, and no studies in which the urinary tract and the male genital tract of leopard geckos could be visualized. Likewise, the visualization of the urogenital tract in the mentioned lizard species should be compared, and its sonographic parameters evaluated with those of preliminary studies or extended to recognize a differentiation from pathological findings.

## 2 Materials and Methods

For this study, a total of 42 lizards, divided into eight male and seven female bearded dragons, six male and nine female leopard geckos, and six male and six female chameleons (divided into seven veiled chameleons and five panther chameleons), were included. The animals were from the patient population of the Clinic for Birds and Reptiles at the University of Leipzig and were examined as part of clinical diagnostics. Approval from the institution's ethics committee was not required for this study as it involved clinical cases with client‐owned animals. All procedures were conducted in accordance with the ethical guidelines for the use of animals in clinical research and included informed consent from the client. The lizards showed a wide age and weight range (1–14.75 years and 35–437 g). Lizards exhibiting overt abnormalities in the urogenital tract were excluded from the study, or only in cases where such abnormalities were deemed to be clinically inconspicuous.

### 2.1 Ultrasonography

Sonographic examinations were performed on each animal by one author (N. K.). Ultrasound examinations were performed using an ultrasound machine (LOGIQ S7 XDclear2.0, GE Ultrasound Korea, Seongnam, Gyeonggi, Korea) and its 8–18 MHz hockey‐stick transducer and 6–15 MHz linear transducer. The hockey‐stick transducer was always preferred, but if the organs were too large to be fully visualized by this transducer, the larger linear transducer was used. As the examinations were carried out in a physiological posture wherever possible and fixation was only necessary for very agile lizards, none of the animals had to be sedated or anaesthetized.

Bearded dragons and leopard geckos were examined in the prone position and fixed in the shoulder area and on the hind limbs if necessary. Docking was first performed from the dorsal side and then from the ventral side. Chameleons were restrained at the head and hind limbs for the examination and examined from the left side, whereby an upright position of the animals was aimed for. The pelvic area was kept free for the transducer. To ensure adequate coupling, a prewarmed ultrasound gel was applied to the skin in the pelvic area and the caudal coelomic region. First, the kidneys and then the gonads were imaged in longitudinal and cross‐section, with and without color Doppler. Sagittal and transverse images and video recordings were taken, stored, and analyzed at a later date. The transducer position, organ position, position in relation to other organs, shape, texture, echogenicity, blood flow, length, width, and height were determined for all organs examined. Organ evaluation and size measurements were performed by one author (N. K.) using a DICOM viewer (IntelliSpace Radiology 4.7.1, Philips Healthcare Informatics Inc., Pleasanton, California, USA). The length describes the distance between the cranial and caudal borders, the width between the left and right borders, and the height between the dorsal and ventral borders.

### 2.2 Postmortem Examination

Of the animals examined, one male bearded dragon, one female leopard gecko, and one male and one female panther chameleon had to be euthanized for medical reasons. These were then dissected, whereby the values already measured by ultrasound were checked and compared in situ.

### 2.3 Statistical Analysis

The statistical analysis was carried out by one author (N. K.) using a statistical program (SPSS Statistics, version 28.0). Although the majority of the values were normally distributed, due to the relatively small group population and a few values that were not normally distributed, it was decided to state the results as median and interquartile range (IQR) with a 95% confidence interval. The correlations between all values collected were calculated using the Spearman–Rho correlation coefficient (𝜌), and the results were considered significant at associated *p*‐values of the two‐sided significance level of *p* ≤.05.

## 3 Results

Ten lizards were partially excluded from the study due to the presence of pathological organ changes or poor tissue visualization. This exclusively concerned the genital tract. For example, the gonads of the six female chameleons could not be examined for the study, as all the chameleons had abnormal follicles or the ovaries had already been removed. This was also the case with one bearded dragon and two leopard geckos. The testicles of one bearded dragon could not be fully visualized. One male bearded dragon was completely excluded from the study due to gout. With the exception of the animals mentioned above, all organs could be visualized sonographically. The recorded weights of the respective species and sexes are shown in Table 1.

**TABLE 1:** The weights (g) given in mean (IQR) of eight male and seven female bearded dragons (*Pogona vitticeps*), six male and nine female leopard geckos (*Eublepharis macularius*), and six male and six female chameleons, divided into seven veiled chameleons (*Chamaeleo calyptratus*) and five panther chameleons (*Furcifer pardalis*).

Species	Total	Males	Females
Bearded dragon	324.5 (121)	316.0 (133)	333.0 (133)
Leopard gecko	62.0 (14)	65.0 (18)	55.0 (14)
Chameleon			
Total	133.0 (31)	144.5 (137)	118.0 (43)
Veiled chameleon	131.0 (95)		
Panther chameleon	136.0 (40)		

### 3.1 Kidneys

**FIGURE 1:**
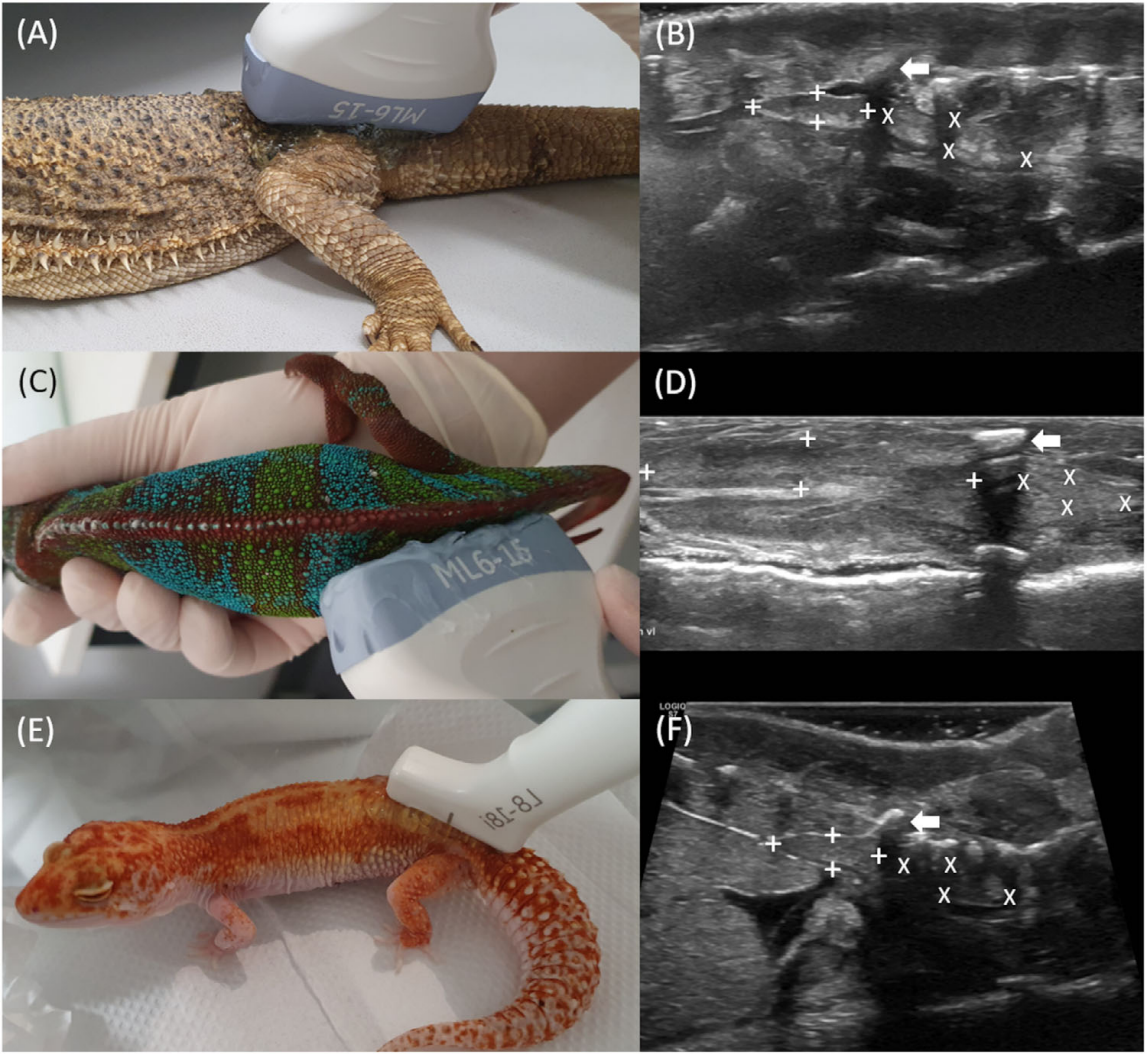
Transducer positions for the renal ultrasound and the corresponding ultrasound images in different lizard species. The cranial pole (boundaries marked by crosses) and the caudal pole (boundaries marked by Xs) are separated by the sound extinction of the hip bone (left arrow). A, Parasagittal renal sound in a bearded dragon with a 6–15 MHz linear transducer. The center is located dorsally at the level of the hip joint. B, Parasagittal ultrasound image of the left kidney of a bearded dragon. The caudal pole is not always clearly visible. C, Lateral kidney ultrasound of a panther chameleon with a 6–15 MHz linear transducer. The center is located laterally about one centimeter in front of the pelvis. D, Lateral ultrasound image of the left kidney of a panther chameleon. The caudal part is clearly smaller than the cranial part. E, Parasagittal kidney ultrasound in a leopard gecko with an 8–18 MHz linear transducer. The center is located dorsally at the level of the hip joint. F, Parasagittal ultrasound image of the left kidney of a leopard gecko. The caudal pole is partially not clearly visible.

**FIGURE 2:**
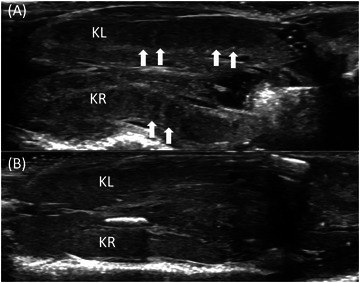
Comparison between male and female kidneys of veiled chameleons in ultrasound. A 12 MHz linear transducer was used in each case. A, Left (KL) and right (KR) kidney of a male veiled chameleon. The sexual complexes appear as hypoechogenic striations (arrow above). B, Left (KL) and right (KR) kidney of a female veiled chameleon. The kidney tissue is homogeneous.

**FIGURE 3:**
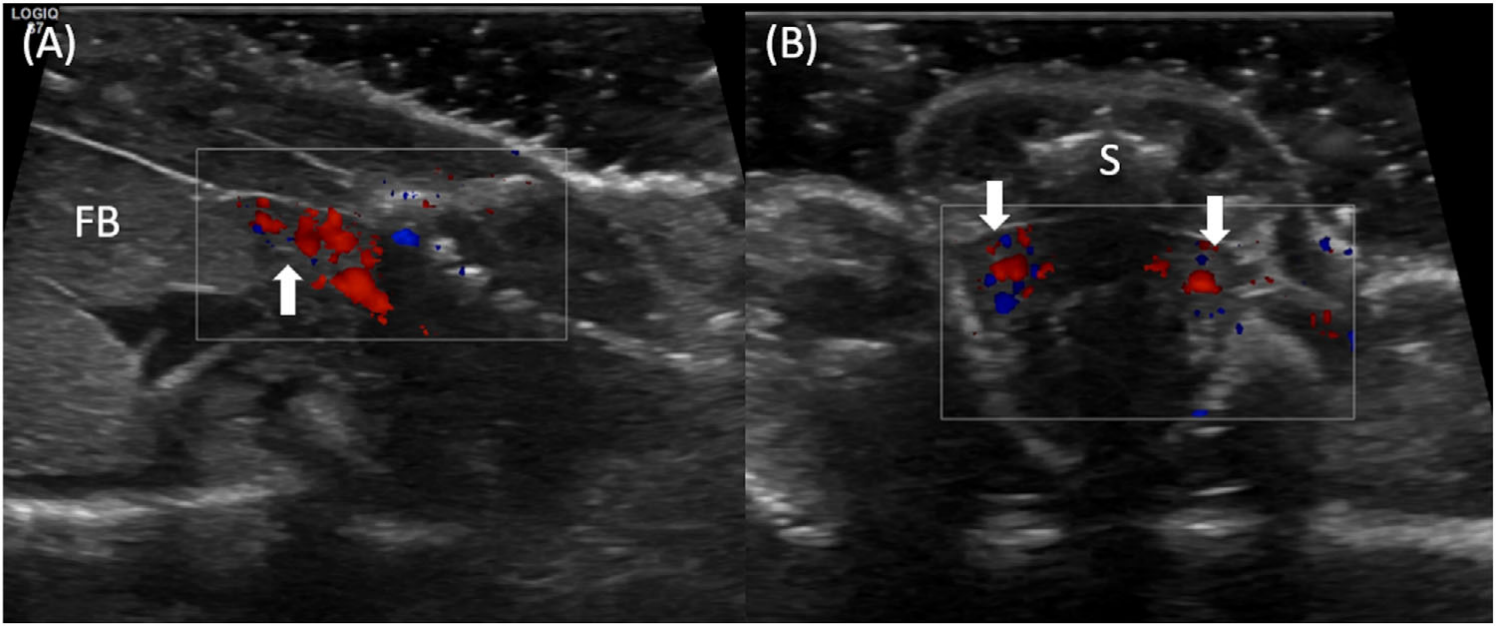
Ultrasound images of the pelvic region of a female leopard gecko using an 18 MHz linear transducer. A, Parasagittal section of the right cranial renal pole (top arrow). Using color Doppler, the renal blood flow can be visualized, and the kidney can be differentiated from the adjacent fat body (FB). B, Transverse section of the left and right kidney (arrows below). Both show good blood flow. The acoustic shadow of the spine (S) can partially obscure parts of the kidneys.

In all lizards examined, the kidneys were found in the caudodorsal coelomic region and partly in the dorsal pelvic canal, ventrolateral to the vertebral column and dorsal to the intestine. The best visualization of the kidneys in bearded dragons and leopard geckos in the parasagittal section was achieved when the transducer was positioned dorsally, centrally above the hip joints, in chameleons correspondingly laterally and slightly shifted cranially (Figure 1). To better visualize the caudal renal poles in some animals, it was sometimes necessary to turn the transducer slightly medially, as the kidneys are connected caudally and become narrower. Only in one female bearded dragon was better visualization achieved ventrally. For a transverse section, the transducer was rotated by 90° around its axis from this position and adjusted cranially or caudally to find the best coupling. Since the spine causes an acoustic shadow in this coupling, which can obscure a larger part of the kidneys, better images could be achieved by angling the transducer slightly laterally. This setting was better than ventral coupling in 7 of 14 (50%) bearded dragons and 12 of 15 (80%) leopard geckos. In the chameleons, the lateral attachment was best in all animals. The shape of the kidneys was elongated and narrow in all animals in the parasagittal section. In the transverse section, bearded dragons and leopard geckos showed mostly (11 of 14 bearded dragons; 78.6%; 13 of 15 leopard geckos; 86.7%) a roundish kidney or, less often, (3 of 14 bearded dragons; 21.4%; 2 of 15 leopard geckos; 13.3%) an oval kidney. In the case of the chameleon, the renal morphology in the transverse section was found to be oval in six of the twelve subjects (50%), while the remaining six chameleons (50%) exhibited a bean‐shaped renal morphology. In the case of male bearded dragons, the otherwise homogeneous tissue in the central to ventral area of the kidneys showed hyperechogenic structures, some of which were strung together like chains. Female bearded dragons, on the other hand, showed a homogeneous kidney texture. The kidneys of leopard geckos were also homogeneous, except for one female in which a hyperechogenic midline was visible. In the chameleons, the kidneys of male animals showed heterogeneous coarse blotching and striation and partial granulation (Figure 2), whereas female chameleons all had a homogeneous kidney texture. The echogenicity of the kidneys in bearded dragons was isoechogenic to the musculature and hypoechogenic to the adipose tissue in all animals. In leopard geckos, it was isoechogenic to more hypoechogenic than the adipose tissue, which made differentiation from the adipose tissue difficult in some cases. In chameleons, it was more isoechogenic than hyperechogenic than muscle and more hypoechogenic than adipose tissue. If it is difficult to differentiate between isoechogenic tissues, a color Doppler can be used to visualize the renal blood flow (Figure 3).

The size measurements of the kidneys are listed in Table 2. Other parts of the urinary tract could not be shown or could not be shown clearly. The respective strong correlations between the kidney values of the mentioned lizard species are listed in Table 3.

**TABLE 2:** The size measurements (mm) given in mean (IQR) of the kidneys of eight male and seven female bearded dragons (*Pogona vitticeps*), six male and nine female leopard geckos (*Eublepharis macularius*), and six male and six female chameleons, divided into seven veiled chameleons (*Chamaeleo calyptratus)* and five panther chameleons *(Furcifer pardalis)*.

		Total			Males			Females		
Species	Kidney	Length	Width	Height	Length	Width	Height	Length	Width	Height
Bearded dragon	Left	29.25 (6.09)	6.08 (1.28)	3.85 (0.70)	29.40 (6.25)	6.05 (0.60)	3.97 (1.03)	29.05 (6.60)	6.65 (1.95)	3.60 (0.63)
	Right	29.85 (4.84)	6.15 (1.30)	3.65 (0.65)	30.30 (5.5)	6.05 (0.65)	3.87 (1.00)	29.40 (5.90)	6.65 (1.80)	3.47 (0.53)
Leopard gecko	Left	15.35 (1.55)	3.75 (1.00)	2.67 (0.47)	16.03 (0.67)	4.10 (0.86)	2.85 (0.47)	15.05 (0.80)	3.75 (1.08)	2.43 (0.45)
	Right	15.20 (1.45)	3.75 (1.15)	2.47 (0.47)	15.98 (0.97)	4.25 (0.94)	2.77 (0.30)	15.10 (0.70)	3.55 (0.95)	2.33 (0.37)
Chameleon	Left	39.95 (2.60)	4.00 (0.68)	10.45 (2.01)	40.55 (2.33)	4.23 (0.75)	11.23 (3.08)	39.63 (5.58)	3.60 (0.64)	9.58 (2.72)
	Right	39.90 (3.34)	4.03 (1.13)	9.77 (2.68)	40.10 (2.20)	4.50 (1.24)	10.95 (3.34)	38.18 (5.01)	3.78 (0.71)	9.45 (3.28)
Veiled chameleon	Left	39.95 (5.70)	3.75 (0.85)	11.03 (2.13)						
	Right	40.45 (5.80)	4.15 (1.30)	10.57 (2.57)						
Panther chameleon	Left	39.95 (2.40)	4.15 (0.77)	9.83 (3.35)						
	Right	39.90 (1.55)	4.00 (0.95)	9.30 (3.03)						

**TABLE 3:** The respective Spearman–Rho correlation (𝜌) and two‐sided significance level (*p*) between the kidney values of eight male and seven female bearded dragons (*Pogona vitticeps*), six male and nine female leopard geckos (*Eublepharis macularius*), and six male and six female chameleons (divided into seven veiled chameleons (*Chamaeleo calyptratus)* and five panther chameleons *(Furcifer pardalis)*).

			Bearded dragon					Leopard gecko					Chameleon				
			Kidney left		Kidney right			Kidney left		Kidney right			Kidney left		Kidney right		
			Width	Height	Length	Width	Height	Width	Height	Length	Width	Height	Width	Height	Length	Width	Height
Kidney left	Length	𝜌	.486	.525	.855^**^	.487	.726^**^	.541^*^	.657^**^	.875^**^	.659^**^	.869^**^	−.178	−.235	.526	−.002	.035
		*p*	.078	.054	.000	.078	.003	.037	.008	.000	.008	.000	.581	.462	.079	.996	.914
		*N*	14	14	14	14	14	15	15	15	15	15	12	12	12	12	12
	Width	𝜌		0.053	.662^**^	.982^**^	.107		.470	.305	.816^**^	.470		.795^**^	.224	.789^**^	.497
		*p*		.858	.010	0.000	.716		.077	.270	0.000	.077		.002	.484	.002	.100
		*N*		14	14	14	14		15	15	15	15		12	12	12	12
	Height	𝜌			.609^*^	.048	.746^**^			.539^*^	.521^*^	.718^**^			.287	.704^*^	.804^**^
		*p*			.021	.869	.002			.038	.047	.003			.366	.011	.002
		*N*			14	14	14			15	15	15			12	12	12
Kidney right	Length	𝜌				.632^*^	.561^*^				.494	.795^**^				.574	.594^*^
		*p*				.015	.037				.061	0.000				.051	.042
		*N*				14	14				15	15				12	12
	Width	𝜌					.108					.681^**^					.511
		*p*					.713					.005					.089
		*N*					14					15					12

* The correlation is significant at the .05 level (two‐sided).

** The correlation is significant at the .01 level (two‐sided).

### 3.2 Testes

**FIGURE 4:**
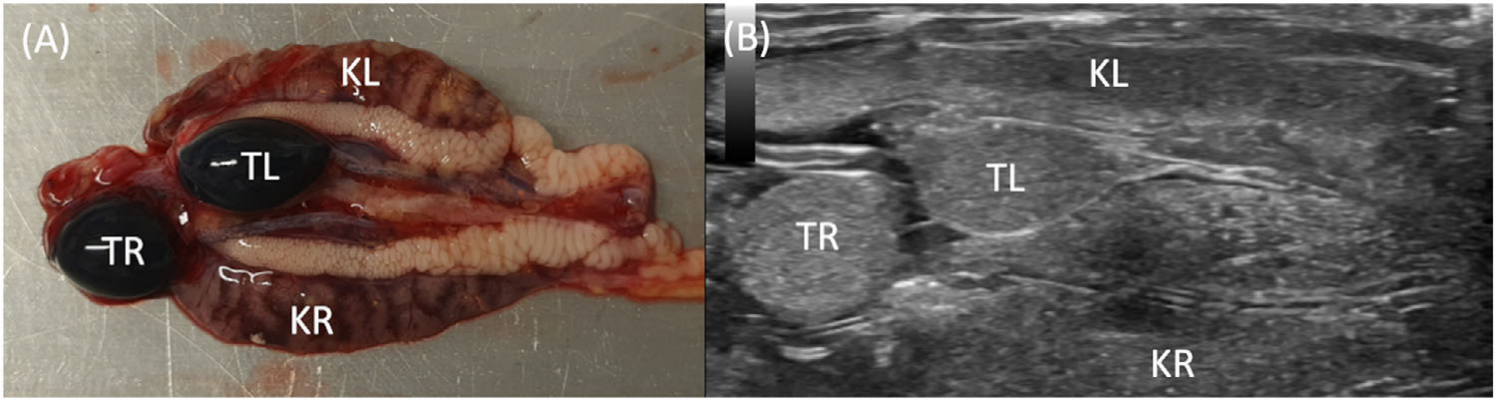
Male urogenital tract in chameleons. A, Isolated urogenital tract of a male panther chameleon. The right testis (TR) is located further cranially than the left testis (TL). They are located cranioventral between the left (KL) and right (KR) kidney.

In all lizards examined, the testes were located at the beginning of the last third of the dorsal coelomic cavity, ventrolateral to the vertebral column and dorsal to the gastrointestinal tract. In chameleons, the testes were located cranioventral to the kidneys, and the right testis was about one third to a whole testicular length further cranial than the left testis (Figure 4).

**FIGURE 5:**
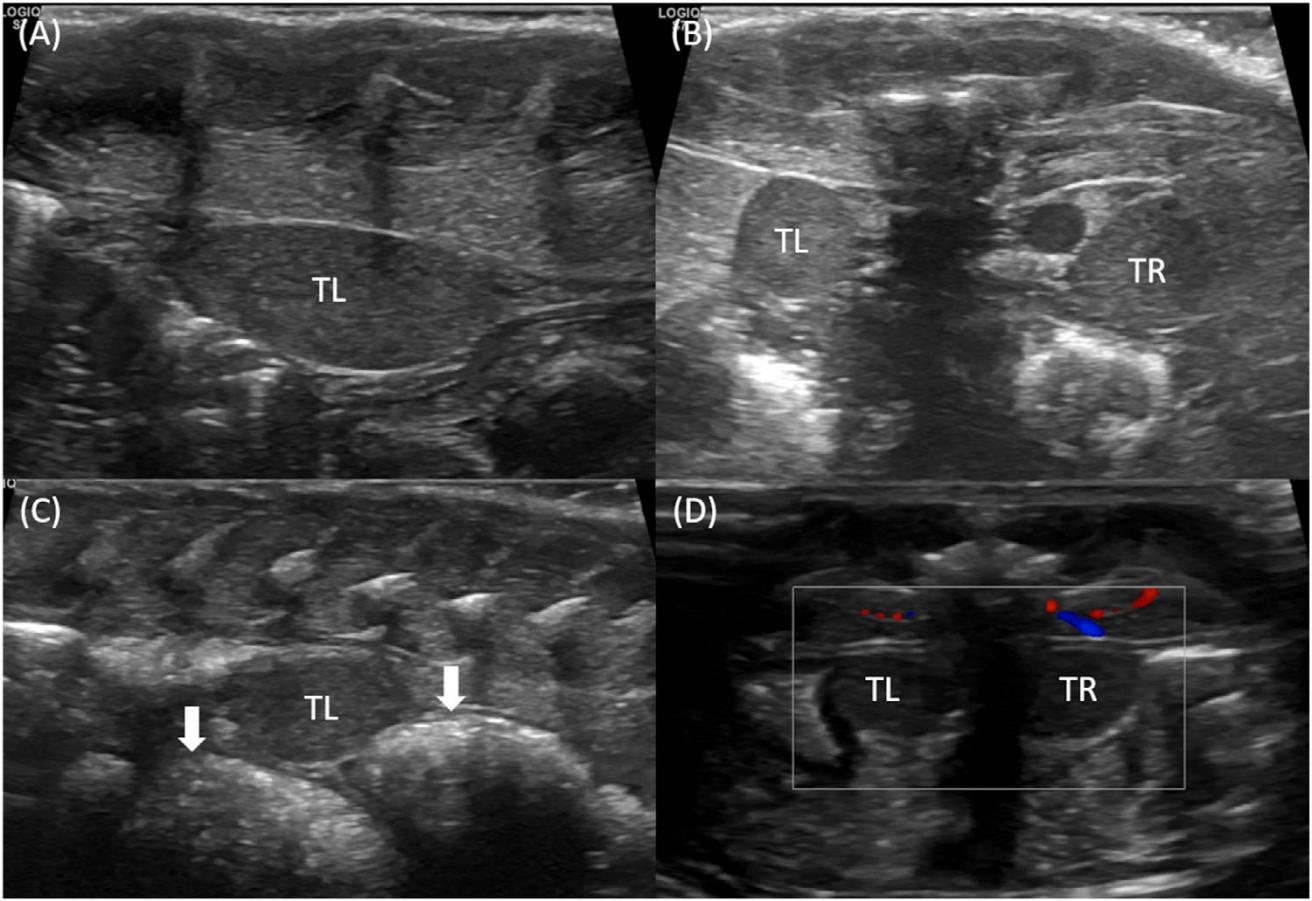
Sonographic imaging of the testes of a bearded dragon and a leopard gecko from the dorsal side using a 15–18 MHz linear transducer. A, The left testis (TL) of a bearded dragon. The tissue is homogeneous, and the shape is oval in longitudinal section. B, Same animal as in (A). Cross‐section of the left and right (TR) testis. The shape is rather roundish. C, The left testis of a leopard gecko. The gastrointestinal tract (arrow below) can be obstructive when viewed ventrally. D, The same animal as in (C). Cross‐section of the left and right testis. Color Doppler shows no blood flow in the testicular tissue.

In bearded dragons and leopard geckos, the testes could be visualized in the parasagittal section from the dorsal side directly next to the vertebral column, or from the ventral side at the same height, and in the transverse section by placing the transducer in the middle of the vertebral column, or slightly shifted laterally if the vertebral column covered parts of the testes (Figure 5). In bearded dragons, the coupling was better from the ventral side in 2 of 6 (33%) and from the dorsal side in 4 of 6 (66%). In leopard geckos, it was better from the ventral side in 1 of 6 (16.7%) and from the dorsal side in 5 of 6 (83.3%). Chameleons were all examined laterally. The testes can be partially obscured by the lungs or parts of the gastrointestinal tract. The shape of the testes was oval in all lizards in the parasagittal section. In the transverse section, they were oval in 3 of 6 (50%) and round‐oval in 3 of 6 (50%) bearded dragons, round in all leopard geckos, and round‐oval in all chameleons.

The testes had a large hyperechogenic capsule in all bearded dragons and a homogeneous texture in 5 of 6 (83.3%). In one animal (16.7%), the testes had a heterogeneous texture with large hyperechogenic structures. In leopard geckos, the testicular texture was homogeneous in 5 of 6 (83.3%) and heterogeneous in 1 of 6 (16.7%), with hyperechogenic granules. The testes of the chameleons had a homogeneous texture and a hyperechogenic capsule.

The testes of bearded dragons had a low to medium echogenicity, and the testes of leopard geckos had a low echogenicity. In chameleons, the testes had low echogenicity except for the largest and heaviest animal (437 g), in which the echogenicity was medium to high. Blood flow to the testicular tissue was either absent or weak in all lizards using color Doppler. Other parts of the male genital tract, such as the vas deferens, could be partially detected but could not be clearly assigned (Figure 4). The size measurements of the testicles are listed in Table 4.

**TABLE 4:** The size measurements (mm) given as median (IQR) of the testicles of eight male bearded dragons (*Pogona vitticeps*), six male leopard geckos (*Eublepharis macularius*), and six male chameleons (divided into two veiled chameleons (*Chamaeleo calyptratus)* and four panther chameleons *(Furcifer pardalis)*.

	Testis	Length	Width	Height
Bearded dragon	Left	12.93 (2.35)	7.43 (1.38)	6.42 (1.48)
	Right	11.65 (2.47)	7.68 (1.91)	6.18 (1.38)
Leopard gecko	Left	8.23 (0.46)	4.55 (0.70)	3.60 (0.88)
	Right	8.23 (1.44)	4.20 (1.00)	3.72 (0.98)
Chameleon	Left	10.00 (0.33)	6.05 (1.61)	6.48 (0.73)
	Right	10.00 (0.74)	5.85 (1.00)	6.28 (1.33)

There was a strong positive correlation in bearded dragons between weight and left testicular length *(𝜌* = 1.00; *p* <.001) and left and right testicular height *(𝜌* = 0.943; *p* <.005).

### 3.3 Ovaries

Inactive ovaries were found in bearded dragons and leopard geckos in the area from the middle to the beginning of the last third of the dorsal coelomic cavity, ventrolateral to the vertebral column and dorsal to the intestine, where they could always be recognized as a collection of roundish follicles in clusters. If vitellogenic follicles were recognizable, their extensions reached into the ventral coelom, and they were located lateral to the intestine.

**FIGURE 6:**
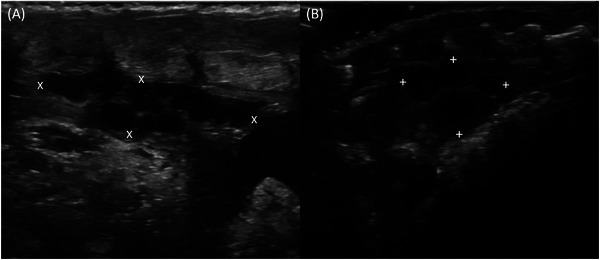
Parasagittal ultrasound images of ovaries, displayed with an 18 MHz linear transducer. Coupling from the dorsal side. A, ovary (X) of a bearded dragon. The previtellogenic follicles are anechogenic. B, ovary (+) of a leopard gecko, dorsally adjacent to the vertebral column and ventrally adjacent to the gastrointestinal tract.

In bearded dragons, the ovaries could be visualized in the parasagittal section from the dorsal side directly next to the vertebral column (Figure 6), or from the ventral side at the same height and in the transverse section by placing the transducer laterally to the vertebral column. In bearded dragons, the ovaries were better visualized ventrally in 3 of 6 animals (50%) in the parasagittal section and 5 of 6 (83.3%) in the transverse section. In leopard geckos, visualization was better from the dorsal side in all animals examined. The follicles in bearded dragons all showed a weak to strong hypo‐ to hyperechogenic border, a homogeneous structure, and an anechogenic echogenicity. In 3 out of 6 of the animals, some of the follicles were also centrally delimited, so that the center appeared anechogenic and the periphery hypoechogenic.

The follicles in leopard geckos all showed a weak hypoechogenic boundary, a homogeneous structure, and an anechogenic echogenicity. In addition, 2 of 7 leopard geckos had follicles with a central demarcation, which was represented by an anechogenic to hypoechogenic center and a hyperechogenic periphery. One leopard gecko had follicles with a ventral demarcation, which were mostly hyperechogenic and only anechogenic in the ventral area. Apart from the ovaries with follicles, no other components of the female genital tract could be visualized. The size measurements of the ovaries are listed in Table 5.

**TABLE 5:** The size measurements (mm) as median (IQR) of the ovaries of seven female bearded dragons (*Pogona vitticeps*) and nine female leopard geckos (*Eublepharis macularius*).

	Ovary	Length	Width	Height	Follicle size	Follicle count
Bearded dragon	Left	14.93 (11.35)	11.33 (8.69)	7.03 (4.40)	1.1–13.6	7–27
	Right	15.78 (8.00)	12.53 (8.00)	7.88 (5.42)	1.1–13.9	9–18
Leopard gecko	Left	9.65 (7.75)	6.30 (7.10)	6.80 (5.20)	1.5–14.9	3–5
	Right	11.80 (6.30)	7.00 (7.15)	7.60 (7.17)	1.2– 15.7	2–7

*Note*: Range of follicle size in mm and number of follicles.

There was a strong positive correlation in leopard geckos between the weight and the number of follicles in the left ovary *(𝜌* = .756; *p* <.049). In contrast, strong negative correlations were found between weight and right ovary length *(𝜌* = −.786; *p* <.036), right ovary width *(𝜌* = −.786; *p* <.036), and right ovary height *(𝜌* = −.821; *p* <.023).

## 4 Discussion

In this study, the kidneys, testes, and ovaries (except chameleons) of male and female bearded dragons, leopard geckos, panther chameleons, and veiled chameleons could be visualized sonographically. In comparison to previous studies [7, 8, 9], the kidneys of these animals could be visualized in full length, although partially interrupted by acoustic shadows. In addition, male leopard geckos and their kidneys and testes were also examined. However, data on the ovaries of female chameleons could not be collected.

The kidneys of bearded dragons and leopard geckos were best visualized from the dorsal side in the parasagittal section and the dorsal side in the transverse section in 50% of the bearded dragons and 80% of the leopard geckos. Since the patient can remain in a physiological body posture with dorsal coupling, and it is therefore less stressful, this variant should also be preferred. In chameleons, on the other hand, lateral coupling always produced the best results. The renal echogenicity in bearded dragons is consistent with the results of previous studies [8], while in chameleons, it was found to be more hypoechogenic compared with adipose tissue [9]. Furthermore, a sexual dimorphism in the form of chain‐like hyperechogenic structures in male bearded dragons and a heterogeneous coarse spotting, striation, and partial granulation in male chameleons could be recognized in the kidney texture. These could belong to the sexual complex, which hypertrophies during the reproductive season [10], but were found in all male bearded dragons and chameleons regardless of the season. By keeping them in a terrarium without an artificially induced seasonal change in light and temperature, as some keepers still do, the sexually active phase could be prolonged or permanently maintained. There was also a difference between the kidney heights of males and females, which has already been described for bearded dragons [8]. The sizes of the kidneys of bearded dragons were similar to those already described [8]. In chameleons, only the kidney width could be compared with previously published values, which also corresponded approximately to these, as in previous studies, the kidney length was not fully recorded, and the height was not recorded at all [9]. Comparative data on the sonographic visualization of kidneys in leopard geckos were not available and could therefore not be compared.

The testes could be fully visualized and examined in all but one bearded dragon. In 66% of the bearded dragons and 83.3% of the leopard geckos, they were easier to assess from the dorsal side, whereas in all chameleons, from the lateral side. The testicular texture of one bearded dragon was consistent with previous results, but the majority had a homogeneous texture [8]. The texture and echogenicity of chameleons’ testes, on the other hand, were comparable in all but one animal. This was an exceptionally large and heavy animal, which means that the increase in echogenicity may also be due to an increased body fat content. As with the kidneys, there were no comparative data on the sonographic visualization of the testicles in leopard geckos. A difference in testicle size between left and right testicles was observed in all species (right smaller than left). In bearded dragons, the mean values of the testicle lengths were smaller than those of comparative studies and the widths in between [8]. The panther chameleons examined had longer testicles than comparative studies, as did the veiled chameleons, which also had a slightly smaller right testicle width [9].

The ovaries of all chameleons, one bearded dragon, and two leopard geckos could not be adequately examined due to a state of distress. In all other animals, the ovaries and their functional bodies could be fully visualized. No eggs were present in any of the animals examined. In the leopard geckos, visualization was most successful from the dorsal side, in the bearded dragons, in the parasagittal section at 50% and the transverse section at 83.3% from the ventral side. This could be related to the different body sizes and bone thicknesses, and to a partial dorsal coverage of the ovaries by the lungs. In addition, leopard geckos tended to show stronger defensive behavior with increased handling and ventral attachment. The follicles of bearded dragons were similar to those described in previous studies [8]. In contrast to previous studies, the ovaries could be visualized on both sides in all leopard geckos examined, and the number of follicles was slightly higher on each side. The follicles were also not uniform in all animals [9]. One follicle showed a ventral demarcation, which may have been in the process of transforming into an egg at the time of the study. Heavier animals tended to have smaller ovaries with more follicles, although this correlation was only found on one side in each case. This could be related to the increased energy consumption and the partial lack of food intake during follicle and egg formation, which mobilizes a lot of fat. In both bearded dragons and leopard geckos, smaller, anechogenic functional bodies represented previtellogenic follicles and larger, centrally delimited vitellogenic follicles.

All sonographic size measurements correspond to the organ measurements taken during the dissections. With the appropriate high‐frequency transducers and a little experience, even very small structures such as the kidneys of leopard geckos can be visualized and assessed. With further advances in technology, there may be even better imaging options in the future, for example, to be able to visualize other parts of the male and female genital tract more accurately.

## List of Author Contributions

### Category 1

- Conception and design: Klützow, Schmidt
- Acquisition of data: Klützow
- Analysis and interpretation of data: Klützow

### Category 2

- Drafting the article: Klützow
- Revising article for intellectual content: Klützow, Schmidt

### Category 3

- Final approval of the completed article: Klützow, Schmidt

## Conflicts of Interest

The authors declare no conflicts of interest.

## EQUATOR Network Disclosure

No EQUATOR network checklist was used.

## Data Availability

The data that support the findings of this study are available from the corresponding author upon reasonable request.
